# A Tale of Two States: Pluripotency Regulation of Telomeres

**DOI:** 10.3389/fcell.2021.703466

**Published:** 2021-07-08

**Authors:** Clara Lopes Novo

**Affiliations:** ^1^The Francis Crick Institute, London, United Kingdom; ^2^Imperial College London, London, United Kingdom

**Keywords:** telomeres, pluripotency, phase separation, chromatin, nuclear architecture, epigenetics

## Abstract

Inside the nucleus, chromatin is functionally organized and maintained as a complex three-dimensional network of structures with different accessibility such as compartments, lamina associated domains, and membraneless bodies. Chromatin is epigenetically and transcriptionally regulated by an intricate and dynamic interplay of molecular processes to ensure genome stability. Phase separation, a process that involves the spontaneous organization of a solution into separate phases, has been proposed as a mechanism for the timely coordination of several cellular processes, including replication, transcription and DNA repair. Telomeres, the repetitive structures at the end of chromosomes, are epigenetically maintained in a repressed heterochromatic state that prevents their recognition as double-strand breaks (DSB), avoiding DNA damage repair and ensuring cell proliferation. In pluripotent embryonic stem cells, telomeres adopt a non-canonical, relaxed epigenetic state, which is characterized by a low density of histone methylation and expression of telomere non-coding transcripts (TERRA). Intriguingly, this telomere non-canonical conformation is usually associated with chromosome instability and aneuploidy in somatic cells, raising the question of how genome stability is maintained in a pluripotent background. In this review, we will explore how emerging technological and conceptual developments in 3D genome architecture can provide novel mechanistic perspectives for the pluripotent epigenetic paradox at telomeres. In particular, as RNA drives the formation of LLPS, we will consider how pluripotency-associated high levels of TERRA could drive and coordinate phase separation of several nuclear processes to ensure genome stability. These conceptual advances will provide a better understanding of telomere regulation and genome stability within the highly dynamic pluripotent background.

## Introduction

Eukaryotic genomes are dynamic, non-randomly organized structures within the nucleus. A complex and highly hierarchical three-dimensional network of structures organizes chromatin into active/inactive compartments, membraneless bodies, lamina associated domains, protein- or RNA-mediated loops, enhancer–promoter contacts, and chromatin regions with differential accessibility. This complex chromatin architecture is established by epigenetic and transcriptional mechanisms and is spatially and temporally tightly regulated, to ensure the maintenance and viability of cellular functions. Chromatin architecture also segregates the large repetitive and gene-poor domains of the genome, like centromeres and telomeres, into constitutive heterochromatin domains characterized by condensed chromatin fibers, high levels of DNA and histone methylation, and transcriptional repression of the underlying DNA sequences (García-Cao et al., 2004; Benetti et al., 2007b; Bickmore and van Steensel, 2013). Constitutive heterochromatin is critical for chromosome segregation and integrity, and changes to the heterochromatic state are commonly associated with aging and cancer (Villeponteau, 1997; Janssen et al., 2018; Valencia and Kadoch, 2019).

Telomeres are nucleoprotein structures formed at the end of chromosomes by the assembly of the shelterin complex (formed by TRF1, TRF2, POT1, TPP1, TIN2 and Rap1) at the TTAGGG telomeric repeats (de Lange, 2005; Martínez and Blasco, 2011). The heterochromatic state is critical for telomere integrity, as deletion of HMTases (SUV39H1/2, SUV4-20H1/2) or DNA methyltransferases (DNMT3A/B, and DNMT1) results in defective telomere function, increased telomere length, and chromosome instability (García-Cao et al., 2004; Gonzalo et al., 2005, 2006). Together with the shelterin complex, the heterochromatic state ensures that telomeres are not recognized as double-strand breaks (DSB), avoiding DNA damage repair and maintaining genome integrity. Paradoxically, in mouse pluripotent embryonic stem cells (mESCs), telomeres adopt a non-canonical epigenetic state that is usually associated with chromosome instability and aneuploidy in somatic cells (Peters et al., 2001; García-Cao et al., 2004), and is characterized by less compaction, low density of histone-methylation and increased TERRA - the telomeric transcripts (Marion et al., 2009; Wong et al., 2009, 2010). Here, we review how the pluripotent nuclear environment of mESCs adopts unique molecular features that contribute or even require a non-canonical telomeric chromatin to safeguard genomic stability (de Lange, 2005; Martínez and Blasco, 2011).

## The Unique Pluripotent Nuclear Environment

mESCs derived from the inner-cell mass (ICM) of early blastocysts retain self-renewal and pluripotent capacity, being able to differentiate into any type of cell. However, the self-renewal and high proliferative capacities expose mESCs to high levels of DNA replication stress (Ahuja et al., 2016). Critically, mutations acquired during early stages of embryonic development must be promptly repaired to prevent chromosomal defects, infertility, or embryonic lethality (Choi et al., 2020). mESCs exploit distinct molecular and biological signatures, like higher proliferative rates, unique cell-cycle composition and checkpoints and better competence for genomic stability maintenance (Boheler, 2009; Boroviak et al., 2015; Ahuja et al., 2016; Vitale et al., 2017).

### The Pluripotent Chromatin Architecture

The chromatin of mESCs has an unusual configuration with open 10 nm chromatin fibers widely dispersed throughout the nucleoplasm, including at constitutive heterochromatin domains (Meshorer et al., 2006; Ahmed et al., 2010; Fussner et al., 2010). Chromatin dispersion is conserved through the cell-cycle, as native mitotic chromosomes purified from mESCs are less condensed than those isolated from other cell-types (Djeghloul et al., 2020), and mESCs contain about 30% less histones than differentiated cells (Karnavas et al., 2014). Dispersed chromatin is also present at the early mouse blastocyst (E3.5) but not in the subsequent stages (E5.5) of development (Ahmed et al., 2010). Thus, this chromatin configuration is considered an architectural hallmark of pluripotency, thought to contribute to pluripotency plasticity by ensuring a transcriptionally permissive and accessible genome (Gaspar-Maia et al., 2011; Cavalli and Misteli, 2013; Hassan-Zadeh et al., 2017).

**FIGURE 1:**
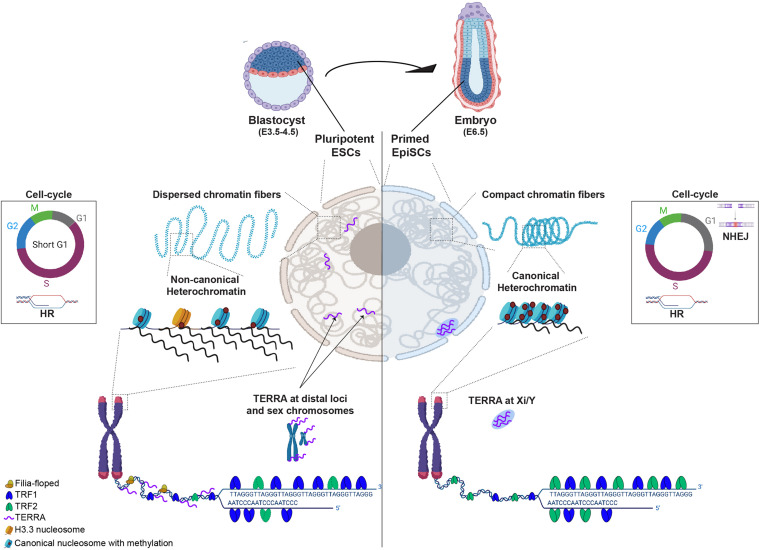
Diagram schematizing differences between pluripotent (cells from blastocyst E3.5-4.5 or ESCs in culture) and cells that exited pluripotency and are primed to differentiate (epiblast at E6.5 embryos). In pluripotent ESCs (left nuclear panel), chromatin fibers are dispersed and heterochromatin is maintained in a non-canonical epigenetic state, characterized by low level of compaction and histone methylation, incorporation of histone H3.3 and high levels of transcription. TRF1, a core shelterin component is highly expressed and TRF2, although present, is not required for telomere protection in ESCs. Filia-floped complexes are recruited to telomeres to mitigate stalled replication forks and telomere transcripts (TERRA) locate at sex chromosomes but also at other distal genomic loci. ESCs have a short G1 phase of the cell-cycle, favoring the use of homologous recombination (HR) to repair DNA damage. In cells that exited pluripotency (in Epiblast of E6.5), chromatin fibers become more dense and heterochromatin adopts a canonical state, characterized by high levels of histone methylation, compaction and transcription silencing. At telomeres, TRF2 becomes crucial for telomere protection and TERRA levels are reduced and redistributed to the inactive sex chromosome. The G1 phase of the cell-cycle becomes longer and cells start using either HR or non-homologous end joining (NHEJ) for DNA repair.

Constitutive heterochromatin rapidly compacts upon mESCs differentiation and in embryo development (Efroni et al., 2008; Wen et al., 2009; Ahmed et al., 2010; Fussner et al., 2010; Figure 1). Forced compaction of heterochromatin domains by disruption of epigenetic regulators (such as *Chd1*, esBAF complex, *Padi4* or H3K9me3 methyltransferases) affects both self-renewal and differentiation potential of mESCs (Meshorer et al., 2006; Gaspar-Maia et al., 2009; Lessard and Crabtree, 2010; Christophorou et al., 2014). Equally, disrupting the pluripotency network by depleting *Nanog*, a key pluripotency transcription factor, impacts the chromatin structure and organization of euchromatin and heterochromatin in mESCs (Novo et al., 2016, 2018). Consistently, forcing heterochromatin decompaction with inhibitors of DNA methyltransferase or histone deacetylases improves the efficiency of somatic cell reprogramming to a pluripotent state (Huangfu et al., 2008; Mikkelsen et al., 2008; Soufi et al., 2012; Sridharan et al., 2013). These findings suggest that changes to the heterochromatin state may be adverse to the pluripotent state.

### Pluripotency and DNA Damage Repair

The pluripotent chromatin architecture is conducive to DNA Damage Repair (DDR), but pluripotent cells adopted strategies to minimize accumulation of DNA mutations and preserve genome stability (Tichy and Stambrook, 2008; Wyles et al., 2014). DDR mechanisms, such as mismatch repair (MMR), base excision repair (BER), nucleotide excision repair (NER), non-homologous end joining (NHEJ), and homologous recombination (HR) repair different types of DNA damage by arresting the cell-cycle at the G1-, S-, or G2/M-phase checkpoints.

In mESCs, cyclins A and CDK1/2 are highly expressed and there is an increased transcription of S-phase genes by hyperphosphorylation of retinoblastoma (Rb), forcing a rapid entry in S-phase (Tsai et al., 2008; Kalaszczynska et al., 2009). This results in an unusually short G1 phase (Tichy et al., 2010), which mESCs compensate for by bypassing the G1/S cell-cycle checkpoint (van der Laan et al., 2013; Kareta et al., 2015; Soufi and Dalton, 2016). Instead, the intra-S and G2 checkpoints are critical for mESCs and consequently the HR pathway is favored for efficiently and accurately repairing DNA double-strand breaks (DSBs) (Tichy et al., 2010; Momčilović et al., 2011; Figure 1). HR proteins, including RAD51, RAD52 and RAD54, are constitutively expressed through the entire cell-cycle (Choi et al., 2017), and the HR process could suffice in efficiently repairing aberrant DNA in mESCs (Yoon et al., 2014; Choi et al., 2017). As mESCs differentiate, expression of HR factors steadily decreases (Choi et al., 2018). Finally, since the strength of the DDR response depends on chromatin compaction levels (Murga et al., 2007), the elevated chromatin accessibility in mESCs can also contribute to a stronger DDR response and genome stability (Murga et al., 2007; Ahuja et al., 2016). In the case of excessive damage, increased mitochondria priming and hyper-sensitivity to apoptosis can remove cells from the mESCs proliferating pool (Roos and Kaina, 2006; Stambrook and Tichy, 2010; Dumitru et al., 2012; Liu et al., 2013).

Heterochromatin is permeable to DNA repair mechanisms (Kallimasioti-Pazi et al., 2018), and so a preference for HR repair in mESCs poses a challenge for these domains due to their repetitive nature and essential function in genome integrity. For example, DNA repair factors like 53BP1 can bind deprotected telomeres, increase their mobility and foster contact with other telomeres, leading to telomere fusions (Dimitrova et al., 2008). However, 53BP1 foci only appear upon irradiation in mESCs, and telomere hyper-recombination is prevented by the telomere-associated protein Rif1 (Dan et al., 2014). Thus, mESCs can exploit alternative mechanisms to compensate for high proliferative rates and to ensure heterochromatin integrity.

### Pluripotency and DNA Replication

DNA replication is essential to the self-renewal and pluripotency capacities of mESCs, whilst conferring an opportunity to alter chromatin with incorporation of new histones or by spatially reorganizing pre-existent histone modifications (McNairn and Gilbert, 2003). Conversely, replication also exposes chromatin to mutations and copy number abnormalities, which could compromise embryonic survival (De and Michor, 2011; Hodgkinson et al., 2012; Schuster-Böckler and Lehner, 2012). Due to the relatively short G1 phase, mESCs are unable to complete DDR before moving to the S-phase (Hyka-Nouspikel et al., 2012; Choi et al., 2017), leading to accumulation of ssDNA gaps and formation of DSBs at stalled replication forks and to accumulation of γH2AX (Chuykin et al., 2008; Banáth et al., 2009; Choi et al., 2020; Blakemore et al., n.d.). Despite rapid proliferation rates and elevated replication stress (Banáth et al., 2009; Ahuja et al., 2016), mESCs have surprisingly low mutation rates. In culture, mESCs display a 1,000-fold lower mutation rate than their isogenically-matched mouse embryonic fibroblasts (Tichy and Stambrook, 2008; Wyles et al., 2014). Therefore, pluripotent mESCs may be more efficient than differentiated cells in resolving replication stress. Indeed, dormant origins can be fired in mESCs to ensure completion of DNA replication under replication stress (Ge et al., 2015). Also, HR factors are constitutively expressed through the cell-cycle, which can facilitate their rapid recruitment to stalled forks in mESCs (Burhans and Weinberger, 2007; Petermann et al., 2010). Indeed, RAD51 depletion in mESCs causes G2/M-phase arrest and replication fork collapse (Petermann et al., 2010). Finally, mESCs use unique protein complexes, like Filia-Floped (a mESC specific regulator of genomic stability and a factor essential for the maternal-zygotic transition, respectively), which scaffold and amplify DDR signaling response at stalled replication forks (Zhao et al., 2015; Figure 1).

In sum, pluripotent cells acquired mechanisms to balance for high proliferative rates without compromising genome integrity, including at heterochromatin domains.

## Pluripotency and Telomeres

### Telomere Length and Pluripotency

Long telomeres are essential for self-renewal and high proliferative capacities in embryogenesis. Two waves of telomere elongation occur during early embryonic development. Through early cleavage stages, parental telomere length is reset and telomeres are elongated by a recombination-based mechanism known as the Alternative Lengthening of Telomeres (ALT) pathway (Schaetzlein et al., 2004; Liu et al., 2007; Varela et al., 2011; Dang-Nguyen et al., 2013). Telomerase activity becomes detectable at the morula-blastocyst transition, when it is thought to stabilize telomere length, and its reverse transcriptase component (TERT) becomes repressed during cellular differentiation, as embryonic development progresses (Holt et al., 1996; Schaetzlein et al., 2004). In humans, telomerase activity is regulated by the alternative splicing of TERT that ensures telomerase repression in somatic cells (Penev et al., 2021).

Short telomeres affect mESCs pluripotency and pose a barrier to an efficient reprogramming process (Zhang et al., 2015). Telomerase deficient mESCs with critically short telomeres are unable to differentiate, as they retain DNA hypomethylation and altered H3K27me3 enrichment at pluripotency promoters, like *Nanog* and *Oct4* (Pucci et al., 2013; Criqui et al., 2020). Telomere length is also influenced by subtelomeric DNA methylation: hypomethylation facilitates recombination-mediated telomere lengthening, while hypermethylation correlates with shorter telomeres (Gonzalo et al., 2006; Yang et al., 2016). These studies strongly support a function for telomere length in regulating the differentiation capacity of mESCs, underscoring the importance of telomere length maintenance in embryonic development.

TBX3, a pluripotency factor required for self-renewal of mESCs and iPSCs (Han et al., 2010; Lu et al., 2011), activates *Zscan4* expression, a 2-cell embryo marker. ZSCAN4 enables heterochromatin decondensation and subtelomeric DNA demethylation in mESCs, promoting telomere elongation by HR (Falco et al., 2007; Zalzman et al., 2010; Macfarlan et al., 2012; Dan et al., 2013; Nakai-Futatsugi and Niwa, 2016) and DNA repair (Akiyama et al., 2015; Eckersley-Maslin et al., 2016; Dan et al., 2017). mESCs expressing *Zscan4* (1–5% of mESCs in serum-culture conditions (Macfarlan et al., 2012)) are characterized by global DNA hypomethylation, histone hyperacetylation, and transcription of heterochromatin domains (pericentromeres, telomeres, and retrotransposons) (Akiyama et al., 2015; Eckersley-Maslin et al., 2016). Interestingly, exogenously induced replication stress in mESCs activates the DNA damage sensor ATR and the transcriptionally signature of 2-cell state, including upregulation of *Zscan4* (Zalzman et al., 2010; Zhang et al., 2016; De Iaco et al., 2017; Hendrickson et al., 2017; Whiddon et al., 2017; Atashpaz et al., 2020). Therefore, an interdependence of distinct pluripotent networks, telomere biology and DNA repair ensure genomic integrity in mESCs.

### Telomere-Associated Proteins

Unexpectedly, telomere binding protein 2, TRF2, a key mediator of telomere protection and core component of the shelterin complex, is dispensable for telomere protection in pluripotent mESCs and in early embryonic development (Markiewicz-Potoczny et al., 2021; Ruis et al., 2021). TRF2 protects and stabilizes telomere structure by binding abnormal DNA conformations that arise at stalled replication forks (like branched DNA, positive DNA supercoils, or G-quadruplexes), suppressing ATM activation and recruiting RTEL1 helicase and other enzymes to remove the blockades (Denchi and de Lange, 2007; Sarek et al., 2016; Mendez-Bermudez et al., 2018). In most cells, TRF2 loss leads to telomere deprotection and fusion via NHEJ (Denchi and de Lange, 2007) but telomeres remain surprisingly protected in mESCs that lack TRF2, despite fully functional ATM and NHEJ pathways (Markiewicz-Potoczny et al., 2021; Ruis et al., 2021). This extraordinary feature of pluripotent telomeres is lost upon differentiation, when TRF2 assumes its fully protective role.

Another core component of the shelterin complex, TRF1, is a direct transcriptional target of the key pluripotent factor *Oct3/4* and is upregulated in pluripotent cells (Boué et al., 2010; Schneider et al., 2013; Figure 1). TRF1 deletion causes embryonic lethality at the blastocyst stage, around E5 (Karlseder et al., 2003; Schneider et al., 2013). Interestingly, TRF2 embryonic lethality occurs much later than TRF1, at E13.5 (Karlseder et al., 2003; Celli and de Lange, 2005), reinforcing the preferential requirement for TRF1 in the pluripotent stages of embryonic development. In most cells, TRF1 promotes DNA replication by blocking HR at telomeres (Karlseder et al., 2003; Sfeir et al., 2009; Porreca et al., 2020). However, TRF1 depletion in induced pluripotent stem cells (iPSCs) leads to genome-wide expression and epigenetic changes through TERRA-mediated Polycomb recruitment to pluripotent and cell-fate genes (Marión et al., 2019).

Thus, pluripotent cells coordinate the pluripotency network, telomere proteins and DNA repair to ensure genome integrity.

### Telomere Chromatin State in Pluripotent Cells

The non-canonical heterochromatin (low density of H3K9me3 and H4K20me3 and increased expression) of pluripotent telomeres may enable access for recombination or telomerase to modulate telomere length (Benetti et al.,2007a,b). As mESCs exit pluripotency, heterochromatin shifts to a canonical state (Azuara et al., 2006; Meshorer and Misteli, 2006). NANOG, a core pluripotent transcription factor, regulates the non-canonical heterochromatin at pericentromeres in mESCs (Novo et al., 2016). Deletion of *Nanog* in mESCs induces a canonical state at pericentromeres, impacts pericentromeric transcription and nuclear architecture and results in genetic instability (Novo et al., 2020). Similarly, interfering with epigenetic factors regulating telomere chromatin in mESCs results in telomere dysfunction and instability (Peters et al., 2001; García-Cao et al., 2004; Benetti et al., 2007b; Dang-Nguyen et al., 2013). Together, these findings suggest that a non-canonical heterochromatin is a requirement for genetic stability in pluripotency.

The ATRX/DAXX chaperone complex deposits histone H3.3, typically associated with active/open chromatin, at telomeres and pericentromeres in mESCs and embryonic germ cells, but not in differentiated cells (Goldberg et al., 2010; Ratnakumar et al., 2012; Clynes et al., 2015; Figure 1). ATRX and H3.3 levels at telomeres decrease as mESCs differentiate (Wong et al., 2009; Lewis et al., 2010), whilst the repressive marks H4K20me3 and H3K9me3 increase (Marion et al., 2009). ATRX knockdown causes telomere dysfunction and up-regulation of TERRA (Goldberg et al., 2010; Wong et al., 2010), and facilitates ALT features at telomeres, in murine cells (Lovejoy et al., 2012). Similarly, H3.3 depletion induces DNA damage and telomeric sister chromatid exchange (Udugama et al., 2015). Importantly, ATRX/DAXX mutations are associated with the ALT mechanism, characterized by telomeres with a relaxed heterochromatin state and high TERRA expression (Lovejoy et al., 2012). However, increased TERRA expression upon ATRX depletion is only observed in murine cells, as ATRX depletion in human cells is insufficient to increase telomeric expression (Episkopou et al., 2014). Despite shared features between ALT + and mESCs telomeres (low H3K9me3 density and compaction and increased TERRA) (Arnoult et al., 2012; Episkopou et al., 2014; Eid et al., 2015), the role of ATRX at telomeres likely depends on cellular context and might also be species-specific.

In human cells, the loss of Tousled-like kinases 1 and 2 (TLK1/2, histone deposition regulators), lead to chromatin decompaction and increased genome accessibility, particularly at heterochromatin domains. Importantly, chromatin decompaction induces heterochromatin expression and ALT features at telomeres (Segura-Bayona et al., 2020), suggesting that telomeres are highly susceptible to chromatin changes. As epigenetic features can impact telomere biology in a cellular-dependent context (Novo et al., 2013), the implications of distinct chromatin states at telomeres need to be further elucidated in different cellular backgrounds.

### Telomere Transcripts in Pluripotent Cells

Telomeric RNA is transcribed by RNA polymerase II moving toward the telomere, from promoters located at subtelomeres, and is composed of G-rich repeats with heterogeneous size (200 bp to several kilobases) (Deng et al., 2012a). In humans, TERRA is transcribed from subtelomeric promoters at most chromosomes ends and stays associated with telomeres (Azzalin and Lingner, 2008; Schoeftner and Blasco, 2008; Zhang et al., 2009; Le et al., 2013; Feretzaki et al., 2019). In mice, TERRA predominantly originates from the pseudoautosomal PAR locus, but TERRA from chromosomes 18q, 2 and X have also been found (López de Silanes et al., 2014; Viceconte et al., 2021). TERRA transcription is sensitive to subtelomeric DNA methylation (Feretzaki et al., 2019). In mESCs, TERRA is enriched at both sex chromosomes and relocates to the heterochromatic sex chromosomes (Y or Xi) during differentiation (Schoeftner et al., 2009; Zhang et al., 2009; Deng et al., 2012a). Despite different origins, and consequently composition, murine and human TERRA share many interacting proteins (including shelterin complex, Bloom helicase, chromatin remodeling factor and DNA replication proteins) (Scheibe et al., 2013; Viceconte et al., 2021), suggesting similar functions. Live-imaging studies showed that TERRA molecules are confined to the telomeric region, forming clusters that may scaffold the nucleation of telomere-associated proteins, as shown for hnRNAP1 or for telomerase (Deng et al., 2012b; Cusanelli et al., 2013; Yamada et al., 2016; Avogaro et al., 2018).

Interestingly, TRF2-TERRA interactions were proposed to mediate telomere heterochromatin in human cells (Deng et al., 2009). As TRF2 appears to be dispensable for mESCs (Markiewicz-Potoczny et al., 2021; Ruis et al., 2021), it is probable that TERRA has a distinct function in murine pluripotent cells. Indeed, most TERRA locates and regulates expression of distal intergenic and intronic regions in the mESCs genome (Chu et al., 2017; Figure 1). However, TERRA depletion in mESCs induces telomere dysfunction, indicating that TERRA is nevertheless important for mouse telomeric integrity (Chu et al., 2017). Importantly, there is conflicting evidence from RNA-FISH vs sequencing-based technologies for TERRA location in mESCs. Thus, elucidating this technical divergence is essential to understand TERRA function and better elucidate how telomere higher-ordered structure impacts genome stability and pluripotency.

## A Golden Thread: Phase-Separation

Phase-separation is based on the spontaneous organization of a solution into two-phases with different densities (Berry et al., 2015; Banani et al., 2017; Boeynaems et al., 2018). The biophysical properties of molecules and their modulation by the surrounding environment enables membraneless compartmentalization and subsequent concentration of biochemical reactions within the cell (Alberti et al., 2019; Gibson et al., 2019). The multivalency of interactions between DNA/RNA molecules and intrinsically disordered regions of proteins can promote liquid–liquid phase separation (LLPS) (Kato et al., 2012; Lin et al., 2015; Hnisz et al., 2017; Langdon et al., 2018). Phase-separation contributes to distinct cellular functions, like stress sensing (Munder et al., 2016; Riback et al., 2017) or increased biochemical kinetics by confining molecules into a compartment (Case et al., 2019). Furthermore, LLPS has been implicated in several nuclear processes, including nucleoli formation, transcription elongation, super-enhancer activity and binding of transcription factors to DNA (Feric et al., 2016; Hnisz et al., 2017; Larson et al., 2017; Strom et al., 2017; Boehning et al., 2018; Boija et al., 2018; Cho et al., 2018; Lu et al., 2018; Sabari et al., 2018; Case et al., 2019; Trivedi et al., 2019; Huo et al., 2020).

Phase-separation has also been proposed to regulate heterochromatin (Larson et al., 2017; Strom et al., 2017; Trivedi et al., 2019; Huo et al., 2020; Novo et al., 2020). The heterochromatin protein 1 (HP1α) is thought to induce LLPS formation at heterochromatin and reinforce the heterochromatic environment by binding to H3K9me3, increasing nucleosome compaction and repressing transcription by exclusion of transcription factors and RNA polymerase (Feng and Michaels, 2015; Sanulli et al.,2019a,b). Similarly, heterochromatin regions interspersed along the chromosome arms can loop and interact in the three-dimensional space (Dernburg et al., 1996; Lee and Karpen, 2017), further supporting the coalescence of multiple condensates into a larger one. CBX2, a subunit of the canonical PRC1 complex responsible for DNA compaction, can also phase-separate both *in vitro* and *in vivo* (Plys et al., 2019; Tatavosian et al., 2019).

Interestingly, the fusion of DNA repair foci into larger clusters was observed in both euchromatin and heterochromatin, which facilitates a rapid but transient recruitment and concentration of repair factors restricted to the damaged region (Aten et al., 2004; Kruhlak et al., 2006; Chiolo et al., 2011; Krawczyk et al., 2012; Aymard et al., 2017; Caridi et al., 2017). Similarly, recruitment of polyADP-ribosylation (PARylation) at DDR foci promotes LLPS (Altmeyer et al., 2015; Duan et al., 2019). Also, the DNA repair protein 53BP1 forms LLPS promoted by non-coding RNA (Binz et al., 2006; Kilic et al., 2019; Pessina et al., 2019). Finally, RAD52 liquid-like condensates formed at different DSB sites can fuse, and mutants unable to form these condensates show limited fusion *in vitro* and increased genome instability *in vivo* (Oshidari et al., 2020). Thus, the ability to phase-separate DNA repair sites could ensure genome stability by restricting access of DDR factors to the damage site (Altmeyer et al., 2015; Patel et al., 2015; Banani et al., 2017). Importantly, DSB repair within constitutive heterochromatin actively decompacts and relocates the damaged locus to the nuclear periphery for HR repair, whilst preventing spurious recombination (Chiolo et al., 2011; Jakob et al., 2011; Janssen et al., 2016; Tsouroula et al., 2016). Whether phase-separation contributes to this mobility, whilst isolating heterochromatin from the surrounding nuclear environment remains to be elucidated.

One hallmark of ALT + cells is telomere clustering at promyelocytic leukemia (PML) bodies, known as ALT-associated PML Bodies (APBs) (Yeager et al., 1999; Heaphy et al., 2011). APBs contain telomeres and many proteins involved in DNA replication, repair, and recombination processes. Interestingly, PML bodies form membraneless organelles by LLPS, mediated by multivalent interactions between SUMO (Small Ubiquitin-like MOdifier) and SIM (SUMO-Interacting Motif) motifs in PML and other proteins (Banani et al., 2017). An elegant study mimicked APBs by engineering polySUMO/polySIM condensates targeted to telomeres in telomerase-positive cells. In the presence of BLM and RAD52, polySUMO/polySIM induce telomere clustering and rapidly recapitulate the ALT phenotype (C-circles, heterogeneous telomere length, and complex telomere structures) (Min et al., 2019). Indeed, telomere clustering seems to depend on the liquid properties of APB condensates, rather than their chemical composition (Zhang et al., 2020).

Pluripotent telomeres may also be able to cluster at PML bodies (Gauchier et al., 2019) but whether phase-separation is involved is still unknown. Also, ATRX recruits HP1a to telomeres in mESCs (Wong et al., 2010; He et al., 2015), where it may mediate HP1a LLPS formation. Interestingly, pericentromeric domains form LLPS condensates in early embryonic development (Strom et al., 2017) and in mESCs (Huo et al., 2020; Novo et al., 2020) but collapse into ‘ordered collapsed globules’ in differentiated cells (Erdel et al., 2020). Thus, the material state of heterochromatin associated with pluripotency seems to facilitate LLPS formation, and may be regulated in different cellular contexts. Importantly, these differences stress the crucial need to characterize phase-separation in many different systems to better understand the mechanisms governing phase-separation.

## Discussion

Telomere maintenance relies on the coordinated crosstalk involving the telomeric structure, TERRA and nuclear processes (such as replication, transcription, repair, etc.). Telomere end protection is critical for genome stability and cell proliferation and the mechanisms involved are fairly conserved across cellular backgrounds and species. Thus, it is striking that a core shelterin component, TRF2, is redundant for telomere protection in pluripotent mESCs. Additionally, as pluripotent cells acquired special features to compensate for the unique nuclear environment, it is paramount to further explore the mechanisms governing telomere maintenance in pluripotent cells.

One of the hallmarks of pluripotent telomeres is high TERRA levels, which may have pluripotent-specific roles, like shown by the regulation of pluripotent gene expression in iPSCs. Furthermore, TERRA molecules can originate from different genomic locations and have different sizes. Thus, regulation of TERRA properties (sequence composition; length; levels) may affect its function by modulating (i) its ability to recruit heterochromatinization factors; (ii) competition with yet unknown cell-specific proteins at telomeres and/or iii) the molecular substrate available for weak multivalent interactions that can affect the material state of telomeres. Strong evidence supports a role for RNA molecules to act as a regulatory elements of LLPS condensate formation, size and constitution (reviewed in Palikyras and Papantonis (2019). Thus, the orchestrated interplay between the pluripotency network, telomeres and DNA repair in mESCs could rely on LLPS to balance the accessible chromatin whilst maintaining genome integrity.

Phase-separation presents an attractive model for harmonizing genome compartmentalization and the diverse biochemical reactions occurring in the nucleus by enabling a spatial and timely unification of nuclear processes through functional concentration of chromatin, RNA/proteins and relevant cellular factors in membraneless compartments. Importantly, as it depends on weak multivalency interactions, phase-separated condensates can dynamically engage in coalescence/fission events to isolate or expose specific chromatin domains, enabling different processes such as replication, transcription or heterochromatin to concomitantly occur within the nuclear environment. Many important questions are left to address and still much to be elucidated, particularly how phase-separation is regulated and how mechanistically promotes cellular functions. Critical open questions are (i) what are the signaling triggers that promote phase-separation; (ii) how the nuclear environment modulates distinct condensates at the same loci and at the same phase of the cell-cycle (for example, transcription vs. heterochromatin aggregates)? New tools that can regulate phase separation in live cells are starting to emerge and will undoubtedly probe cellular functions and the functional possibilities enabled by phase-separation.

## Author Contributions

CN wrote the manuscript and prepared the figure.

## Conflict of Interest

The author declares that the research was conducted in the absence of any commercial or financial relationships that could be construed as a potential conflict of interest.
